# Salinity stress results in ammonium and nitrite accumulation during the elemental sulfur-driven autotrophic denitrification process

**DOI:** 10.3389/fmicb.2024.1353965

**Published:** 2024-02-14

**Authors:** Wenjie Fan, Xuejiao Huang, Jianhua Xiong, Shuangfei Wang

**Affiliations:** ^1^Guangxi University, Nanning, China; ^2^Guangxi Key Laboratory of Agro-Environment and Agro-Product Safety, College of Agriculture, Guangxi University, Nanning, China; ^3^Guangxi Key Laboratory of Environmental Pollution Control and Ecological Restoration Technology, Guangxi Bossco Environmental Protection Technology Co., Ltd., Nanning, China

**Keywords:** sulfur autotrophic denitrification, salinity, nitrite accumulation, ammonium accumulation, microbial communities

## Abstract

In this study, we investigated the effects of salinity on elemental sulfur-driven autotrophic denitrification (SAD) efficiency, and microbial communities. The results revealed that when the salinity was ≤6 g/L, the nitrate removal efficiency in SAD increased with the increasing salinity reaching 95.53% at 6 g/L salinity. Above this salt concentration, the performance of SAD gradually decreased, and the nitrate removal efficiency decreased to 33.63% at 25 g/L salinity. Approximately 5 mg/L of the hazardous nitrite was detectable at 15 g/L salinity, but decreased at 25 g/L salinity, accompanied by the generation of ammonium. When the salinity was ≥15 g/L, the abundance of the salt-tolerant microorganisms, *Thiobacillus* and *Sulfurimonas*, increased, while that of other microbial species decreased. This study provides support for the practical application of elemental sulfur-driven autotrophic denitrification in saline nitrate wastewater.

## 1 Introduction

As anthropological activity increases, an increasing amount of nitrogen-containing wastewater is discharged into the environment, causing eutrophication, which threatens ecosystems, physical health, and ultimately human survival (Jiang et al., 2020; Xu et al., 2022). Nitrate-containing wastewater can cause the quality of water bodies to deteriorate thereby increasing the risk of methemoglobinemia, non-Hodgkin lymphoma, and heart disease in humans (Nuhogl et al., 2002). Consequently, treating nitrate-containing wastewater to meet the emission standards of different industries is essential. In recent years, biological denitrification technology has been identified as an efficient and cost effective method for removing nitrate-nitrogen (${\text{NO}}_{3}^{-}-\text{N}$) from water bodies (Li S. et al., 2020). Biological denitrification technology includes heterotrophic and autotrophic denitrification in accordance with differences in denitrifying microbial carbon sources (Ren et al., 2021). Heterotrophic denitrification (HD) technology has the advantage of a rapid reaction rate and high denitrification efficiency. However, additional carbon is required during the HD process, and a lot of sludge and CO_2_ are produced, which limits the achievement of carbon reduction and neutrality goals (Pang and Wang, 2021). Conversely, autotrophic denitrification, particularly sulfur autotrophic denitrification (SAD), does not require additional carbon sources. In addition, low amounts of sludge and CO_2_ are produced during this process. Therefore, SAD gradually became a promising nitrate-reducing wastewater treatment method (Tian and Yu, 2020).

Salinity has various effects on SAD, which are driven by different reductants. Usually, most nitrate-containing wastewaters such as contaminated groundwater (Zhang et al., 2023), aquaculture wastewater (Ching and Redzwan, 2017), and agricultural production wastewater (Lefebvre and Moletta, 2006) contain high salt concentrations (0–50 g/L). Salinity affects microbial activity, thus influencing the ${\text{NO}}_{3}^{-}-\text{N}$ removal efficiency (Navada et al., 2020; Li et al., 2023). Zhu et al. (2023) found that a salinity of 80 g/L significantly inhibited the growth of *Sulfurimonas* and *Thiobacillus*, thereby decreasing the effectiveness of using bacteria for ${\text{NO}}_{3}^{-}-\text{N}$ removal in the SAD system, with thiosulfate as the reductant. Li et al. (2023) found that anammox and shortcut sulfur autotrophic denitrification (SSADN, with sulfide as the reductant) activity increases with increasing salinity when the salinity was < 2%. When the salinity reached 5%, the nitrogen removal efficiency of this system decreased significantly, and the microbial structure and abundance changed noticeably. Shen et al. (2023) explored the effects of salinity on double short-cut sulfur autotrophic denitrification (DSSADN, using sulfide as the reductant). A salinity of 1.5% strongly stimulated ${\text{NO}}_{3}^{-}-\text{N}$ removal by microorganisms, but once the salinity reached 3% it had an inhibiting effect.

In addition, different types of influent have significant differences in the treatment of pollutants in saline wastewater. Current reactor inlet water types are divided into the sequential batch reactor (SBR) and continuous flow reactor (CFR). SBR has good biomass sedimentation and effective resistance to water and pollutant impacts (Yan et al., 2022), and has been widely used to treat ${\text{NO}}_{3}^{-}-\text{N}$ in saline wastewater (Wang et al., 2020; Zhang et al., 2022). However, compared to the CFR, the SBR has the disadvantage of a complex design, unstable effluent, and the need for regular maintenance (Corsino et al., 2016). For large-scale treatment plants requiring a large amount of continuous wastewater, the CFR operation has higher treatment efficiency and better treatment consistency than the SBR. Zhu et al. (2022) compared the ability of the SBR and the CFR to treat high-salinity wastewater, and the results showed that the CFR exhibited better pollutant removal performance than the SBR. Compared to other reductants in the SAD process, the elemental sulfur-driven SAD process has exhibited low toxicity, low cost, stability, and it is widely used in practical wastewater treatment plants that rely on a continuous inflow (Li Y. et al., 2020; Zhou et al., 2021). Nonetheless, little attention has been paid to the influence of salinity on the SAD process driven by S^0^ continuous flow bioreactors.

In this study, elemental sulfur was used as the reductant in a SAD reactor. NaCl was added to study the effect of salinity on nitrogen transformation. The relationship between salinity and microbial community structure was also analyzed. The results provide a reference for the application of SAD in high-nitrate high-salinity wastewater.

## 2 Materials and methods

### 2.1 Test equipment and operating process

**Figure 1:**
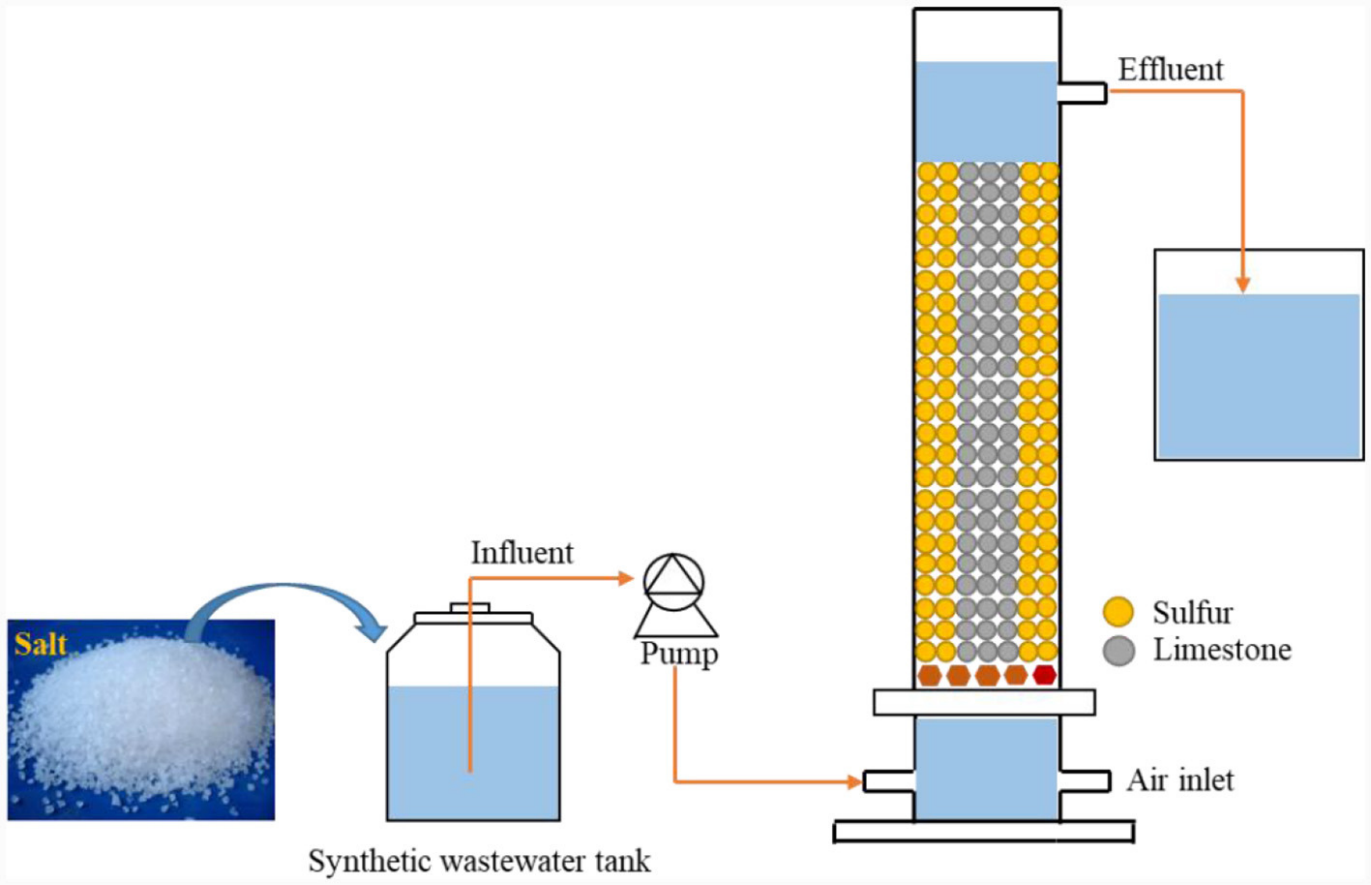
Schematic diagram of the S^0^-driven SAD reactor.

The experimental setup consisted of a cylindrical Plexiglas denitrification bioreactor (outer diameter: 8.6 cm; inner diameter: 7.6 cm; total height: 100 cm; and volume: 4 L) (Figure 1). The reactor was filled with elemental sulfur and limestone particles in a 1:1 volume ratio, and the fillers were 2–3 mm in size. Elemental sulfur served as the reductant (electron donor) in the denitrification process, while limestone regulated the reactor's pH balance. The reactor was continuously fed with water, and wastewater was pumped into the device through a peristaltic pump from the bottom of the reactor. After the reaction was complete, the effluent flowed out near the top of the reactor. The up-flow water intake created anaerobic conditions for microorganismal growth, which helped gases to the escape of gases and ensured interaction between the microorganisms and wastewater. The reactor was wrapped in tinfoil to avoid photosynthetic bacterial propagation. The inoculated sludge (MLSS) was obtained from the anaerobic section-activated sludge of a sewage treatment plant in Nanning, Guangxi, China, with a ~3,000 mg MLSS/L concentration. The sludge inoculation ratio was 20%.

### 2.2 Artificial configuration of synthetic wastewater

The wastewater contained synthetic NO_3_-N derived from stepwise addition of 30 ± 2 mg/L of NaNO_3_ to the influent. Additionally, 210 ± 2 mg/L of ${\text{HCO}}_{3}^{-}$ was added to the influent to provide an inorganic source of carbon. When the SAD reactor operated stably, the volumetric load of nitrogen was controlled so that it remained unchanged, and the salinity of the wastewater was gradually increased from 0 to 25% (Tan et al., 2019; Zhang et al., 2021). The SAD reaction was maintained continuously throughout the experimental process. The hydraulic retention time (HRT) of the reactor was 4 h. Micronutrients were added to maintain microbial growth (1 mL/L), and the nutrient fluid (1 L) contained: 0.12 g ZnSO_4_-7H_2_O, 0.12 g MnCl_2_-4H_2_O, 0.15 g H_3_BO_3_, 0.03 g CuSO_4_-5H_2_O, 0.15 g CoCl_2_-6H_2_O, 0.06 g Na_2_MoO_4_-2H_2_O, and 10 g EDTA (Chauhan et al., 2022). The pH was adjusted to 7.2–7.3 using sodium hydroxide and hydrochloric acid. All dosages were configured to the required concentration. To prevent temperature changes from affecting the system's denitrification, the reactor operated stably at room temperature (25 ± 1°C).

### 2.3 Water quality determination

The reactor effluent was collected every 24 h, and water samples were stored at −20°C. After centrifugation, the supernatant of the water samples were taken for water quality analysis based on the guidelines provided by Eaton (2002) to determine the following water quality indicators. TN concentrations were determined using the alkaline potassium persulfate digestion–UV spectro-photometric method; ${\text{NO}}_{3}^{-}-\text{N}$ using the hydrochloric acid photometry method; nitrite nitrogen (${\text{NO}}_{2}^{-}-\text{N}$) using the N-(1naphthalene)-dia-minoethane spectrophotometry method; ammonium nitrogen (${\text{NH}}_{4}^{+}-\text{N}$) using the indophenol blue method; and sulfate (${\text{SO}}_{4}^{\text{2}-}$) using the barium chromate photometric method; the inlet and outlet water pH was measured with a benchtop pH meter (S220 Micro, Mettler Toledo, Columbus, OH, USA); and salinity was measured using a salinometer (SSM-500, China).

The ${\text{NO}}_{3}^{-}-\text{N}$ and TN removal efficiencies were calculated using the Equation 1:


(1) $$\begin{array}{l} {\text{R}}_{\text{V}}=\left(S_{1}-S_{2}\right)/S_{1}\times 100\% \end{array}$$

where *R*_V_ is the removal efficiency (%), and S_1_ and S_2_ are the initial and final concentrations, respectively. Microsoft Excel 2020 and Origin 2021 were used for statistical and graphical analyses.

### 2.4 EPS extraction and analysis

Biofilm-attached packing samples were collected from the reactors on days 15, 30, 45, 60, and 75 to determine the extracellular polymer (EPS). Biofilms were collected using a previously described sampling method (Hao et al., 2022). EPS was extracted from the biofilm using a heating method (Li and Yang, 2007). The amount was calculated as follows: EPS = protein (PN) content + polysaccharide (PS) content. The PS and PN contents were determined using the phenol–sulfuric acid and Lowry methods, respectively (Shi et al., 2020).

### 2.5 Scanning electron microscope observations

Biofilm-attached packing samples were collected from the reactors on days 15, 30, 45, 60, and 75 and then fixed by adding a 2.5% glutaraldehyde solution (Petcharat and Benjakul, 2018). The processed samples were rinsed three times with phosphate buffer (pH 7.0), followed by stepwise dehydration in ethanol (30–50–70–80–90–95%) for 15 min each. Finally, they were rinsed twice for 20 min with 100% ethanol. After vacuum freeze-drying, gold was sprayed on the samples and they were viewed using a biological scanning electron microscope (SEM; Hitachi SU8010, Hitachi Ltd., Tokyo, Japan) (Huang et al., 2022).

### 2.6 Microbial community analysis

Biofilm samples were collected on days 15, 30, 45, 60, and 75, respectively, and stored at −80°C for 16S rRNA determination. Genomic DNA was extracted from the biological samples using a DNA extraction kit (E.Z.N.A.^®^ Soil DNA Kit, Omega Bio-tek, Norcross, GA, USA). Meiji Biomedical Technology Co. (Shanghai, China) performed the polymerase chain reaction amplification of the16S rRNA gene, Illumina MiSeq sequencing, and data analytics. The amplification primers were bacterial 338F (5′-ACTCCTACGGGAGGCAGCAG-3′) and 806R (5′-GGACTACHVGGGTWTCTAAT-3′) for the V3–V4 regions (Xu et al., 2016). The specific analyses were conducted online on the website Majorbio I-Sanger platform (www.i-Sanger.com).

## 3 Results and discussion

### 3.1 Startup of the SAD system

**Figure 2:**
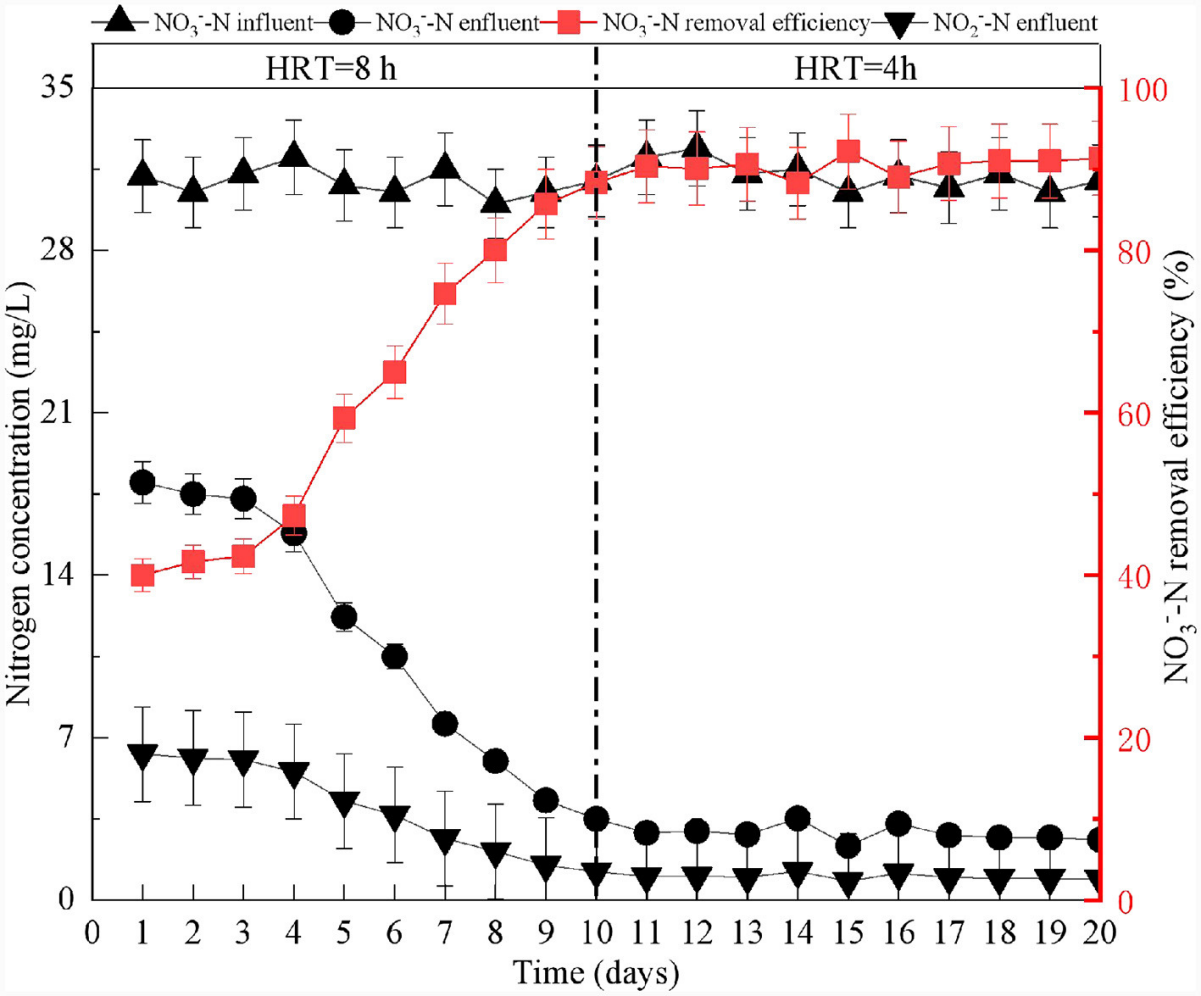
Startup of S^0^-driven autotrophic denitrification reactor.

**Figure 3:**
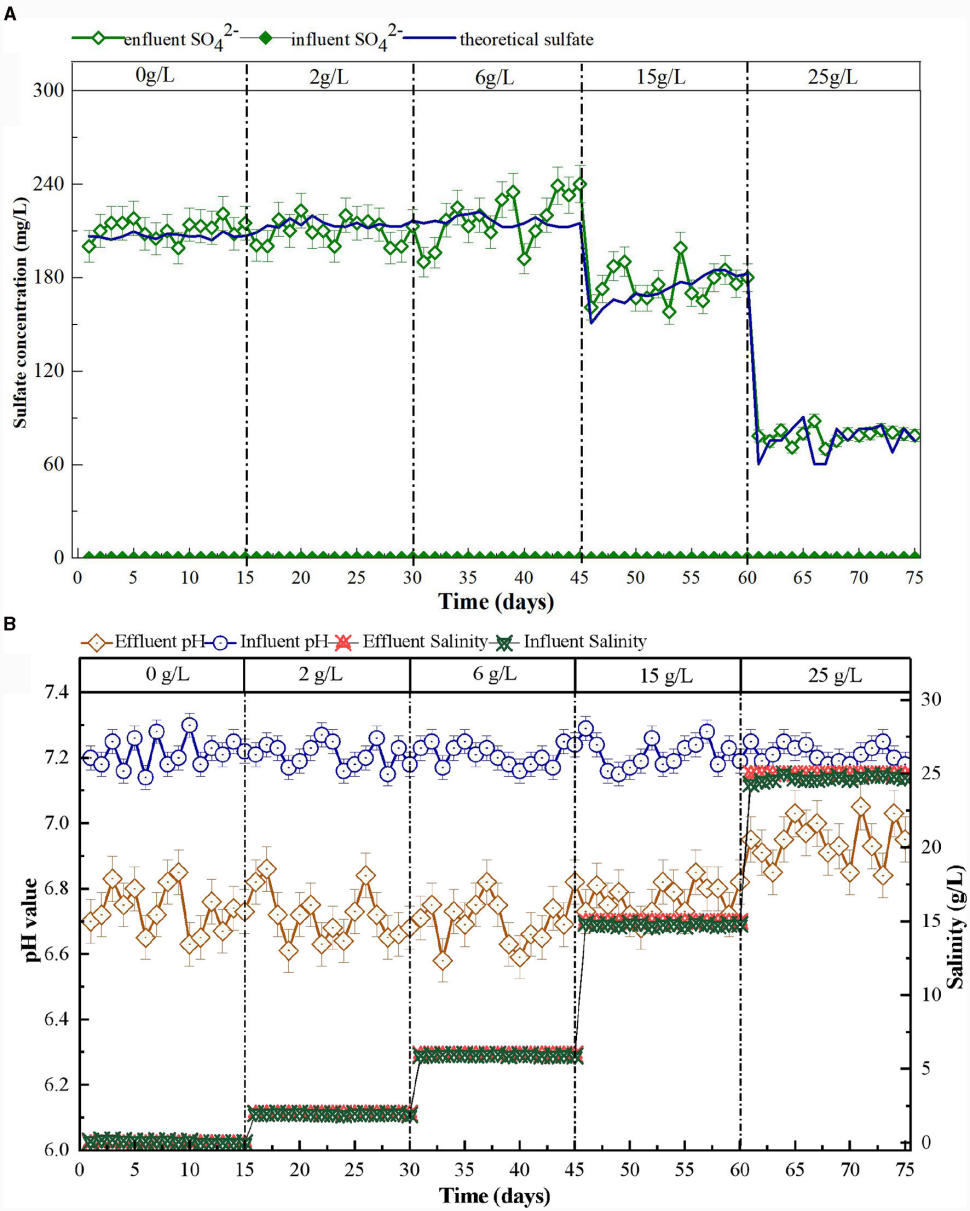
Variation of sulfate concentration **(A)**, pH and salinity **(B)** in S^0^-driven SAD system at different salinity.

At the beginning of the reactor start-up, the effluent concentrations of ${\text{NO}}_{3}^{-}-\text{N}$ decreased gradually. After 20 days of reactor operation, the ${\text{NO}}_{3}^{-}-\text{N}$ removal efficiency increased to 90%, and no ${\text{NO}}_{2}^{-}-\text{N}$ was detected (Figure 2). These results indicated that the reactor had successfully started (Shen et al., 2023; Zhu et al., 2023). Previous research has demonstrated that ${\text{SO}}_{4}^{\text{2}-}$ production confirms the presence and degree of SAD (Chen et al., 2018; Li et al., 2022). According to Equation (2), for every 1 mg/L of ${\text{NO}}_{3}^{-}-\text{N}$ consumed, 7.83 mg/L of ${\text{SO}}_{4}^{\text{2}-}$ is produced (Capua et al., 2019). Using the calculations, the theoretical ${\text{SO}}_{4}^{\text{2}-}$ yield should be 214 mg/L when 27.3 mg/L of ${\text{NO}}_{3}^{-}-\text{N}$ is removed in the SAD system. The observed effluent concentration of ${\text{SO}}_{4}^{\text{2}-}$ was 219 mg/L (Figure 3A). This result demonstrated that ${\text{NO}}_{3}^{-}-\text{N}$ removal in the system was mainly via sulfur autotrophic denitrification. Owing to the addition of limestone to neutralize the H^+^ produced in the SAD process (Guo et al., 2022), the pH value of the effluent decreased slightly but stabilized at approximately pH 6.7 (Figure 3B). The reactions were as follows.


(2) $$\begin{array}{l} {\text{S}}^{0}+0.88{\text{NO}}_{3}^{-}+0.34{\text{H}}_{2}\text{O}+0.38{\text{HCO}}_{3}^{-}+0.02{\text{CO}}_{2}+0.08{\text{NH}}_{4}^{+}\\ \;\to 0.08{\text{C}}_{5}{\text{H}}_{7}O_{2}\text{N}+0.82{\text{H}}^{+}+0.44{\text{N}}_{2}+{\text{SO}}_{4}^{\text{2}-} \end{array}$$

**Figure 4:**
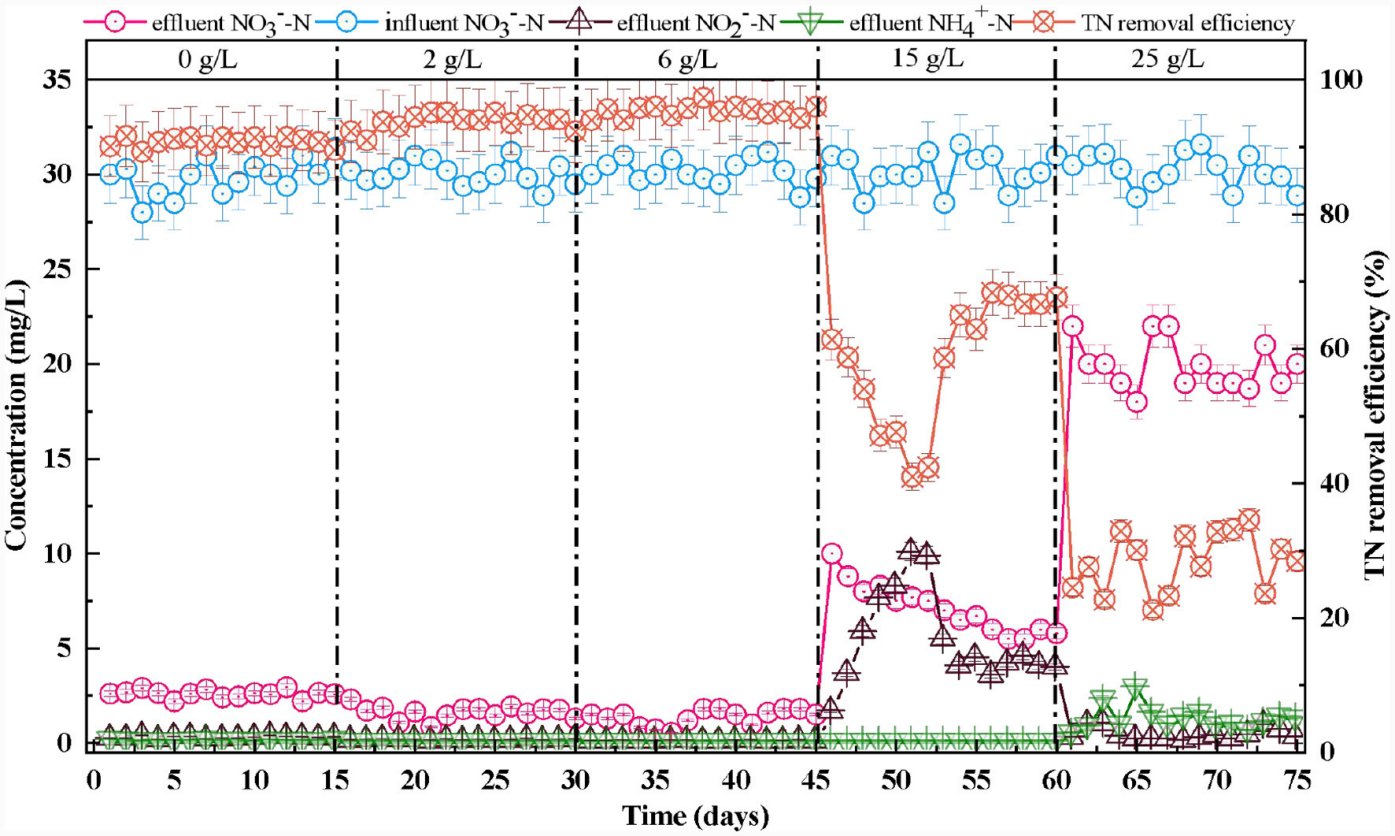
Effect of salinity on nitrogen transformation in S^0^-driven SAD system.

After the reactor was stabilized, the ${\text{NO}}_{3}^{-}-\text{N}$ removal efficiency remained >90%, corresponding to a TN removal efficiency of 90.5%. This left almost undetectable ${\text{NO}}_{2}^{-}-\text{N}$ and ${\text{NH}}_{4}^{+}-\text{N}$ in the effluent (Figure 4). This illustrated that the microorganisms in the SAD system could efficiently remove ${\text{NO}}_{3}^{-}-\text{N}$ through to almost complete denitrification (Wang S. et al., 2021; Guo et al., 2022).

### 3.2 Effect of salinity on nitrogen and sulfur transformations in SAD system

As the salinity increased from 0 to 6 g/L, the ${\text{NO}}_{3}^{-}-\text{N}$ conversion capability increased continuously (Figure 4). The ${\text{NO}}_{3}^{-}-\text{N}$ and TN removal efficiencies increased to 95.5 and 95.3%, respectively. Moreover, the effluent ${\text{NO}}_{2}^{-}-\text{N}$ and ${\text{NH}}_{4}^{+}-\text{N}$ concentrations also remained undetectable. This phenomenon indicated that the low salinity (2 and 6 g/L) stimulation could enhance ${\text{NO}}_{3}^{-}-\text{N}$ removal. Ding et al. (2023) also reported that 7 g/L salinity could increase the ${\text{NO}}_{3}^{-}-\text{N}$ removal efficiency (from 54.1 ± 12.5% to 74.4 ± 11.53%) in wetlands. This might be because appropriate amounts of salt can promote the enzymatic reactions of the microorganism, maintain their membrane balance, and regulate their cellular osmotic pressure (Bassin et al., 2017). The effluent pH was maintained at ~6.6–6.8, and the effluent ${\text{SO}}_{4}^{\text{2}-}$ concentration increased gradually as the ${\text{NO}}_{3}^{-}-\text{N}$ removal efficiency increased (Figure 3).

When the salinity increased to 15 g/L, the concentration of ${\text{NO}}_{3}^{-}-\text{N}$ in the effluent of the system began to increase gradually and stabilized at 5.5 mg/L (Figure 4). The ${\text{NO}}_{3}^{-}-\text{N}$ removal efficiency initially declined to 66.8% and then increased to 81.7% after the reactor had been running for 15 days. The effluent ${\text{SO}}_{4}^{\text{2}-}$ concentration dropped to 150 mg/L, then increased to ~175 mg/L (Figure 3A). The effluent pH maintained at ~6.6–6.8 (Figure 3B). Therefore, a salinity of 15 g/L would not have an inhibitory effect on functional microorganisms in the SAD system. The reactor would recover once these microorganisms adapted to the high-salt environment (Zhu et al., 2023). The effluent ${\text{NO}}_{2}^{-}-\text{N}$ concentration increased to 10 mg/L then decreased and remained at 4 mg/L during this process (Figure 4). Similarly, Shen et al. (2023) found that gradually increasing salinity to 1.5% promoted ${\text{NO}}_{2}^{-}-\text{N}$ accumulation in the SAD system, probably because the nitrite reductase was sensitive to the increase in salinity. When the salinity continued to increase to 25 g/L, the concentration of ${\text{NO}}_{3}^{-}-\text{N}$ in the effluent continually increased to ~20 mg/L. The corresponding ${\text{NO}}_{3}^{-}-\text{N}$ and TN removal efficiencies decreased to 33.6 and 28.3%, respectively (Figure 4). The effluent ${\text{SO}}_{4}^{\text{2}-}$ concentration dropped to 53 mg/L (Figure 3A). This indicated that a salinity of 25 g/L would significantly inhibit the function of microorganisms in the SAD system. The ${\text{NO}}_{2}^{-}-\text{N}$ concentration in the effluent dropped to 0.1 mg/L, and the ${\text{NH}}_{4}^{+}-\text{N}$ concentration increased to 1 mg/L (Figure 4). This might be because the high salinity stress stimulates dissimilatory nitrate reduction to ammonium (DNRA) (Giblin et al., 2010). The effluent pH therefore increased to 6.8–7.0 (Figure 3B).

### 3.3 Microbial community changes under different salinities

To further explore the influence of salinity on the performance of the SAD system, SEM and microbial community analysis were performed on the filler samples collected at the reactor startup (0 g/L salinity), 2, 6, 15, and 25 g/L salinity levels, respectively.

**Figure 5:**
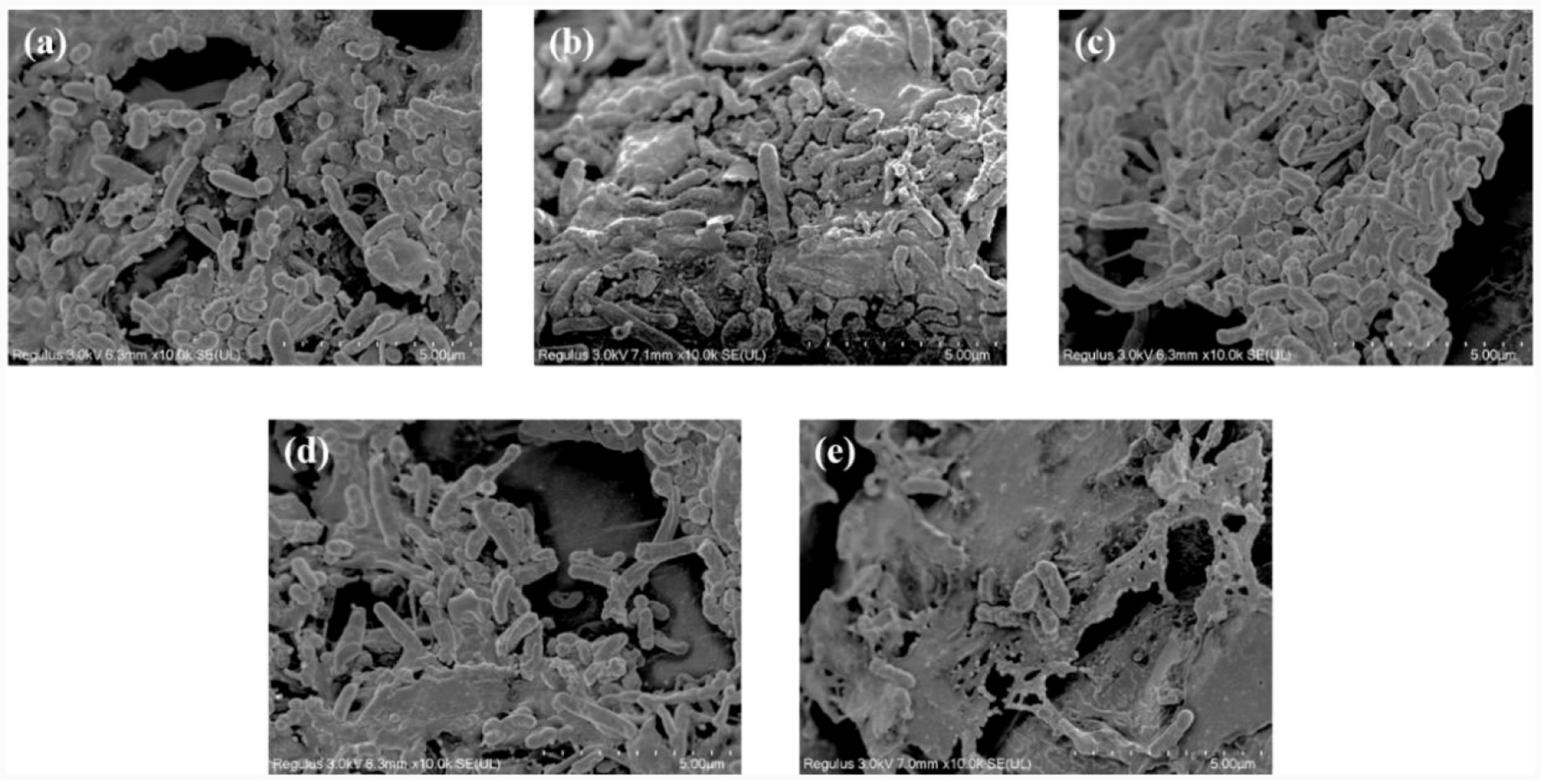
Scanning electron microscope (SEM) images of the filter samples. Salinity of **(a)** 0 g/L, **(b)** 2 g/L **(c)** 6 g/L **(d)** 15 g/L, and **(e)** 25 g/L.

Figures 5a–e shows the structures and microbial morphologies of the filler samples at the different salinities. At 0 g/L salinity, short bacilli and cocci dominated the bacterial morphology (Figure 5a), and their size and morphology were consistent with those of *thiobacillus azotizans*, reported in an earlier study (Guo et al., 2022). When the salinity increased to 2 and 6 g/L, the numbers of short bacilli and spherical bacteria on the filler increased significantly and were attached to the entire surface of the filler (Figures 5b, c). This result indicated that low salinity could stimulate the growth of microorganisms in the system, and promote the removal efficiency of ${\text{NO}}_{3}^{-}-\text{N}$ (Figure 4). However, at salinities of 15 and 25 g/L, the number of microorganisms attached to the filler decreased significantly and were dominated by short bacilli (Figures 5d, e). Similarly, the Chao index increased in low-salinity wastewater (2 and 6 g/L), but significantly decreased in high-salinity wastewater (15 and 25 g/L) compared with non-saline water (Table 1). This indicates that appropriate salinity increases the bacterial community richness in sewage. In contrast, high salinity can kill salt-intolerant microorganisms, thereby reducing community richness (Zhu et al., 2023). The Shannon index decreased and the Simpson index increased as the salinity increased (Table 1). This indicated that some microorganisms in the SAD system could not adapt to the high-salinity environment; however, some salt-tolerant microorganisms continued to increase (He et al., 2020).

**Table 1:** Bacterial community diversity and richness of filter samples.

**Sample**	**Ace**	**Chao**	**Coverage**	**Shannon**	**Simpson**
I	468.518	462.264	0.998	4.287	0.093
II	456.281	475.031	0.998	3.951	0.124
III	450.447	468.501	0.998	3.529	0.283
IV	360.575	351.088	0.998	2.634	0.472
V	264.529	251.667	0.998	2.235	0.823

**Figure 6:**
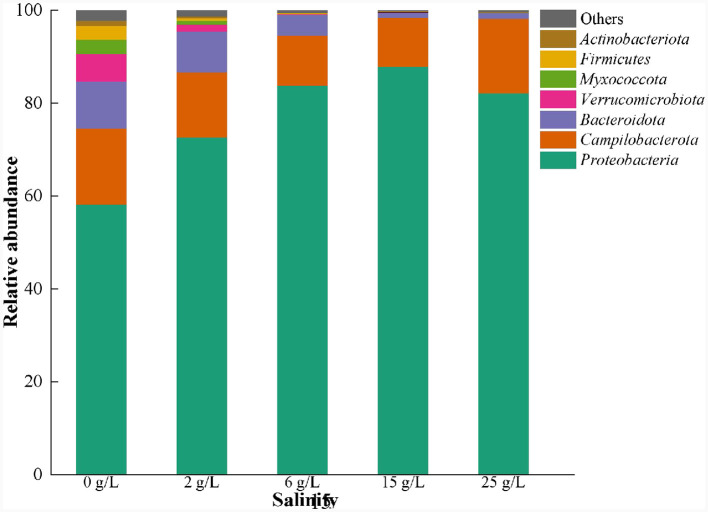
Microbial community changes at phylum level in S^0^-driven SAD system at different salinity.

The dominant phyla in the salt-free SAD system were Proteobacteria (58.2%), Campilobacterota (20.0%), Bacteroidota (10.1%), and Verrucomicrobiota (6.5%) (Figure 6). The relative abundances of Bacteroidota and Verrucomicrobiota decreased significantly with the increase in salinity. However, the abundances of Proteobacteria increased in 0–15 g/L salinity, and then decreased at 25 g/L salinity, indicating initial significant stimulation and subsequent inhibition owing to salt stress (Ding et al., 2023; Shen et al., 2023). In contrast, the abundances of Campilobacterota decreased gradually with the increasing salinity but increased at 25 g/L salinity, indicating that some of the microorganisms in this phyla are salt-tolerant, and they adapt to salinity stress over time (Ohore et al., 2022).

**Figure 7:**
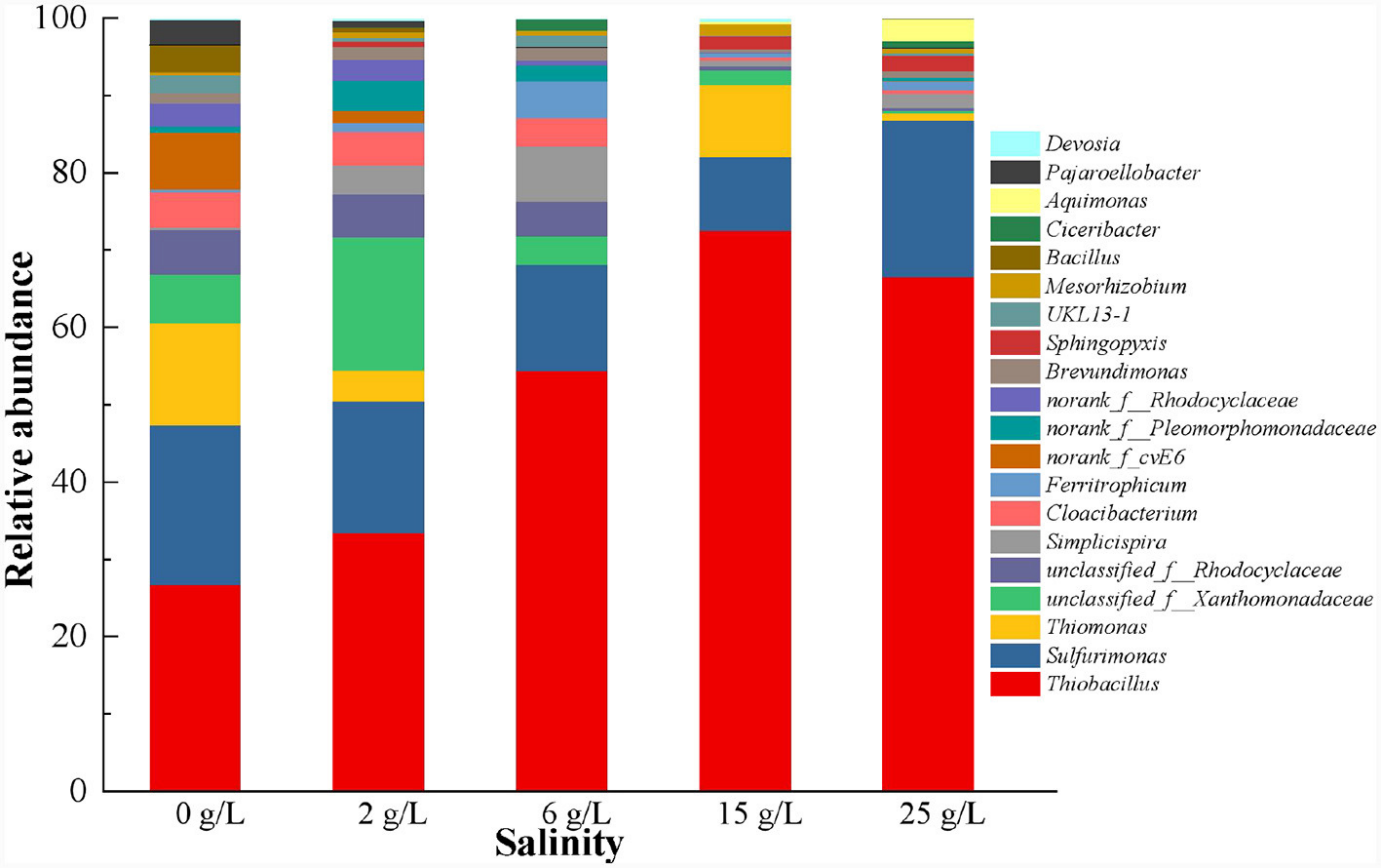
Microbial community changes at genus level in S^0^-driven SAD system at different salinity.

At the genus level, the SOB-related genera *Thiobacillus* (21.1%), *Sulfurimonas* (16.4%), and *Thiomonas* (10.5%), which are commonly present in the SAD system (Blázquez et al., 2019; Polizzi et al., 2022; Yuan et al., 2022; Shen et al., 2023), were detected (Figure 7). *Thiobacillus* was dominant because the SAD system was driven by sulfur (S^0^) (Polizzi et al., 2022), and its abundance increased significantly from 21.1 to 71.1% when the salinity increased from 0 to 15 g/L. However, high salinity (25 g/L) inhibited the growth of *Thiobacillus* with a slight reduction in its abundance. *Sulfurimonas* could barely grow efficiently with sufficient S^2−^ (Polizzi et al., 2022), and was therefore less abundant than the genus *Thiobacillus* in the S^0^-driven SAD system. Moreover, the abundance of *Sulfurimonas* decreased gradually with increasing salinity but increased at 25 g/L salinity, which was similar to the variation trend of the abundance of Proteobacteria (Figure 6). *Sulfurimonas* belongs to Proteobacteria, and is widely distributed in global deep-sea hydrothermal environments (Wang S. S. et al., 2021). Therefore, the genus *Sulfurimonas* in the phylum Proteobacteria might be a salt-tolerance microorganism in the SAD system. *Thiomonas* was highly abundant in 0 and 15 g/L salinity (10.5 and 10.2%, respectively) but decreased to 0.81% in 25 g/L salinity (Figure 7), indicating that it was intolerant to high-salinity environments.

In addition, the abundance of other autotrophic denitrifying bacteria, such as *Ferritropicum* and *Cloacibacterium*, had the ability to remove ${\text{NO}}_{3}^{-}-\text{N}$ from the system (Li Y. et al., 2020; Chauhan et al., 2022) also changed with the increase of salinity. The abundance of *Ferritropicum* increased from 0.31 to 4.47% when the salinity increased from 0–6 g/L, but decreased when the salinity was ≥15 g/L. Song et al. (2020) also found that low salinity (20‰) can promote the growth of *Ferritropicum*. Furthermore, the abundance of *Cloacibacterium* remained unchanged when the salinity increased from 0 to 6 g/L but decreased significantly when the salinity reached 15 and 25 g/L. Moreover, a few heterotrophic bacteria *unclassified_f_Rhodocyclaceae* (4.53%)*, unclassified_f_Xanthomonadaceae* (5.01%), *Cloacibacterium* (3.56%), and *Simplicispira* (0.31%) were detected in the SAD system (Figure 7), and have been identified as denitrifying bacteria in prior studies (Fitzgerald et al., 2015; Si et al., 2021; Liang et al., 2022; Zhang et al., 2023). The abundance of these three genera in the SAD system when the salinity was ≤ 6 g/L was higher than that when the salinity was ≥15 g/L. These results indicated that the heterotrophic bacteria in the SAD system were salt-intolerant. *Unclassified_f_Rhodocyclaceae* and *unclassified_f_Xanthomonadaceae* are dominant bacterial species that use ${\text{NO}}_{2}^{-}-\text{N}$ as an electron acceptor and can be used to reduce ${\text{NO}}_{2}^{-}-\text{N}$, with a higher affinity for ${\text{NO}}_{2}^{-}-\text{N}$ (Hou et al., 2022). When the salinity exceeds 15 g/L, the significant decrease in their abundance may be the reason for the inability to reduce ${\text{NO}}_{2}^{-}-\text{N}$, which resulted in a large ${\text{NO}}_{2}^{-}-\text{N}$ accumulation (Figure 4). Low salt conditions stimulated the growth of some autotrophic and heterotrophic bacteria, resulting in the coexistence of these bacteria in the system. The co-participation of autotrophic and heterotrophic bacteria at low salinity (≤ 6 g/L) promotes the ${\text{NO}}_{3}^{-}-\text{N}$ removal efficiency.

When the salinity was ≥15 g/L, *Thiobacillus* and *Sulfurimonas* that closely related to the DNRA process (Robertson et al., 2016; Lai et al., 2020) occupied a dominant position in SAD (Figure 4). Usually, a correlation between the ammonium flux and salinity strengthens with increasing salinity, but high salinity leads to an inhibition of the denitrification process and enhancement of the DNRA process (Giblin et al., 2010). In this study, high salinity (25 g/L) inhibited denitrification in the SAD system, but *Thiobacillus* and *Sulfurimonas* could reduce ${\text{NO}}_{3}^{-}-\text{N}$ to ${\text{NH}}_{4}^{+}-\text{N}$ via the DNRA pathway, resulting in the accumulation of ${\text{NH}}_{4}^{+}-\text{N}$ (Figure 4).

### 3.4 Effects of salinity on the EPS content and composition

EPS is a self-protective layer of microorganisms, helping to maintain bacterial growth and reactor stability when environmental pressure changes (Han et al., 2020). In this study, we found that *Thiobacillus* and *Sulfurimonas* were the main salt-tolerant microorganisms in the SAD system. Therefore, the EPS was further analyzed to explore why these two genera could tolerate high salinity.

**Figure 8:**
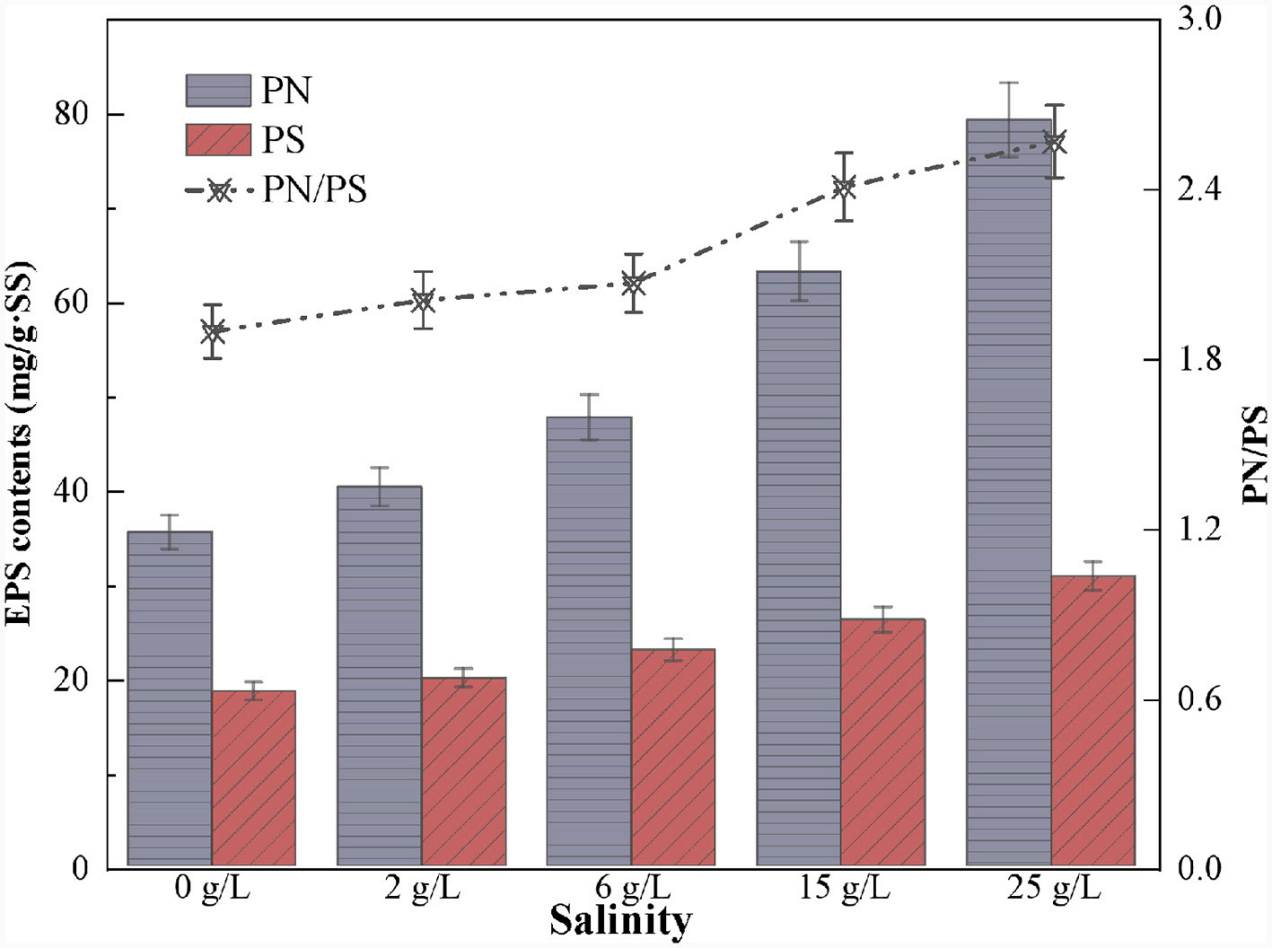
Variation of extracellular polymeric substances content in S^0^-driven SAD system at different salinity.

The results showed that the EPS content gradually increased with increasing salinity (Figure 8), consistent with the phenomenon observed by Song et al. (2016). PN is usually considered a hydrophobic substance crucial for resisting external osmotic pressures and improving sludge performance (Han et al., 2018). Previous studies found that microorganisms in the system secrete more PN under salinity stress (Lin et al., 2022; Sun et al., 2022). This is because the amino and carboxyl groups in PN increase the hydrophobicity of the cell surface, which helps to maintain the structure and stability of the biofilm (Wang et al., 2013; He et al., 2019). In this study, the PN content increased from 35.5 to 79.3 mg/g SS, and the PS content from 18.6 to 30.8 mg/g SS with the contentious increase in salinity (Figure 8). Furthermore, the PN/PS ratio consistency increased from 1.91 in non-saline to 2.57 at 25.0 g/L salinity conditions, suggesting that the PN content exceeded the PS content. Therefore, possibly the dominant strains in the SAD system resisted the high salinity stress by releasing PN.

## 4 Conclusions

Salinities of 2 and 6 g/L promoted the ${\text{NO}}_{3}^{-}-\text{N}$ removal efficiency of the elemental sulfur-driven SAD system, which increased from 91.1 to 94.8% and 95.5%, respectively. However, when the salinity increased to 15 and 25 g/L, the ${\text{NO}}_{3}^{-}-\text{N}$ removal efficiency decreased significantly to 81.7 and 33.7%, respectively. This was accompanied by accumulation of nitrite and ammonium. *Thiobacillus* and *Sulfurimonas* play important roles when the system is in a high salt environment and they release EPS, particularly PN, to resist the salinity.

## Data availability statement

The original contributions presented in the study are publicly available. This data can be found here: https://www.ncbi.nlm.nih.gov; SAMN39477880-SAMN39477884.

## Author contributions

WF: Data curation, Project administration, Visualization, Writing—original draft. XH: Conceptualization, Funding acquisition, Supervision, Writing—review & editing. JX: Funding acquisition, Resources, Supervision, Writing—review & editing. SW: Funding acquisition, Resources, Supervision, Writing—review & editing.

## References

[B1] Bassin J. P. Pronk M. Muyzer G. Kleerebezem R. Dezotti M. Van Loosdrecht M. C. M. (2017). Effect of elevated salt concentrations on the aerobic granular sludge process: linking microbial activity with microbial community structure. Appl. Environ. Microbiol. 77, 7942–7953. 10.1128/AEM.05016-11 21926194 PMC3208997

[B2] Blázquez E. Baeza J. A. Gabriel D. Guisasola A. (2019). Treatment of real flue gas desulfurization wastewater in an autotrophic biocathode in view of elemental sulfur recovery: microbial communities involved. Sci. Total Environ. 657, 945–952. 10.1016/j.scitotenv.2018.12.037 30677960

[B3] Capua F. Pirozzi F. Lens P. Esposito G. (2019). Electron donors for autotrophic denitrification. Chem. Eng. J. 362, 922–937. 10.1016/j.cej.2019.01.069

[B4] Chauhan S. Sharma V. Varjani S. Sindhu R. Bhargava P. C. (2022). Mitigation of tannery effluent with simultaneous generation of bioenergy using dual chambered microbial fuel cell. Bioresour. Technol. 351:127084. 10.1016/j.biortech.2022.127084 35358671

[B5] Chen D. Wang H. Yang K. Ma F. (2018). Performance and microbial communities in a combined bioelectrochemical and sulfur autotrophic denitrification system at low temperature. Chemosphere 193, 337–342. 10.1016/j.chemosphere.2017.11.017 29149709

[B6] Ching Y. C. Redzwan G. (2017). Biological treatment of fish processing saline wastewater for reuse as liquid fertilizer. Sustainability 9:1062. 10.3390/su9071062

[B7] Corsino S. F. Campo R. Di Bella G. Torregrossa M. Viviani G. (2016). Study of aerobic granular sludge stability in a continuous-flow membrane bioreactor. Bioresour. Technol. 200, 1055–1059. 10.1016/j.biortech.2015.10.065 26526094

[B8] Ding Y. Wang Y. Gu X. Peng Y. Sun S. He S. (2023). Salinity effect on denitrification efficiency with reed biomass addition in salt marsh wetlands. Bioresour. Technol. 371, 128597. 10.1016/j.biortech.2023.128597 36632851

[B9] Eaton A. D. (2002). “State environmental protection administration of China,” in Standard Methods for the Examination of Water and Wastewater. Beijing: Environmental Science Press, 254–279.

[B10] Fitzgerald C. M. Camejo P. Oshlag J. Z. Noguera D. R. (2015). Ammonia-oxidizing microbial communities in reactors with efficient nitrification at low-dissolved oxygen. Water Res. 70, 38–51. 10.1016/j.watres.2014.11.041 25506762 PMC4564296

[B11] Giblin A. E. Weston N. B. Banta G. T. Tucker J. Hopkinson C. S. (2010). The effects of salinity on nitrogen losses from an oligohaline estuarine sediment. Estuar. Coasts 33, 1054–1068. 10.1007/s12237-010-9280-7

[B12] Guo G. Li Z. Chen L. Ling Q. (2022). Advances in elemental sulfur-driven bioprocesses for wastewater treatment: from metabolic study to application. Water Res. 213:118143. 10.1016/j.watres.2022.118143 35149365

[B13] Han F. Ye W. Wei D. Xu W. Du B. Wei Q. (2018). Simultaneous nitrification-denitrification and membrane fouling alleviation in a submerged biofilm membrane bioreactor with coupling of sponge and biodegradable PBS carrier. Bioresour. Technol. 270, 156–165. 10.1016/j.biortech.2018.09.026 30218931

[B14] Han F. Zhang M. Shang H. Liu Z. Zhou W. (2020). Microbial community succession, species interactions and metabolic pathways of sulfur-based autotrophic denitrification system in organic-limited nitrate wastewater. Bioresour. Technol. 315:123826. 10.1016/j.biortech.2020.123826 32682266

[B15] Hao W. Li Q. Liu P. Han J. Duan R. Liang P. (2022). A new inoculation method of sulfur autotrophic denitrification reactor for accelerated start-up and better low-temperature adaption. Sci. Total Environ. 823:153657. 10.1016/j.scitotenv.2022.153657 35122857

[B16] He Q. Chen L. Zhang S. Chen R. Wang H. (2019). Hydrodynamic shear force shaped the microbial community and function in the aerobic granular sequencing batch reactors for low carbon to nitrogen (C/N) municipal wastewater treatment. Bioresour. Technol. 271, 48–58. 10.1016/j.biortech.2018.09.102 30261336

[B17] He Q. Wang H. Chen L. Gao S. Zhang W. Song J.. (2020). Robustness of an aerobic granular sludge sequencing batch reactor for low strength and salinity wastewater treatment at ambient to winter temperatures. J. Hazard. Mater. 384:121454. 10.1016/j.jhazmat.2019.121454 31668764

[B18] Hou R. Yuan R. Chen R. Zhou B. Chen H. (2022). Metagenomic analysis of denitrifying phosphorus removal in SBR system: comparison of nitrate and nitrite as electron acceptors. Chem. Eng. J. 446:137225. 10.1016/j.cej.2022.137225

[B19] Huang J. L. Jiang D. H. Wang M. S. Huang X. J. (2022). Highly selenite-tolerant strain *Proteus mirabilis* QZB-2 rapidly reduces selenite to selenium nanoparticles in the cell membrane. Front. Microbiol. 13:862130. 10.3389/fmicb.2022.862130 35479612 PMC9037631

[B20] Jiang M. Zheng X. Chen Y. (2020). Enhancement of denitrification performance with reduction of nitrite accumulation and N_2_O emission by *Shewanella oneidensis* MR-1 in microbial denitrifying process. Water Res. 169:115242. 10.1016/j.watres.2019.115242 31706124

[B21] Lai J. Cheng M. Huang R. Yu G. Chong Y. Li Y.. (2020). Mechanism of ammonium sharp increase during sediments odor control by calcium nitrate addition and an alternative control approach by subsurface injection. Environ. Res. 190:109979. 10.1016/j.envres.2020.109979 32745537

[B22] Lefebvre O. Moletta R. (2006). Treatment of organic pollution in industrial saline wastewater: a literature review. Water Res. 40, 3671–3682. 10.1016/j.watres.2006.08.027 17070895

[B23] Li S. Zhang H. Huang T. Ma B. (2020). Aerobic denitrifying bacterial communities drive nitrate removal: performance, metabolic activity, dynamics and interactions of core species. Bioresour. Technol. 316:123922. 10.1016/j.biortech.2020.123922 32758920

[B24] Li X. Yuan Y. Dang P. Li B. L. Huang Y. Li W.. (2023). Effect of salinity stress on nitrogen and sulfur removal performance of short-cut sulfur autotrophic denitrification and anammox coupling system. Sci. Total Environ. 878:162982. 10.1016/j.scitotenv.2023.162982 36958564

[B25] Li X. Y. Yang S. F. (2007). Influence of loosely bound extracellular polymeric substances (EPS) on the flocculation, sedimentation and dewaterability of activated sludge. Water Res. 41, 1022–1030. 10.1016/j.watres.2006.06.037 16952388

[B26] Li Y. Liu L. Wang H. (2022). Mixotrophic denitrification for enhancing nitrogen removal of municipal tailwater: contribution of heterotrophic/sulfur autotrophic denitrification and bacterial community. Sci. Total Environ. 814:151940. 10.1016/j.scitotenv.2021.151940 34843783

[B27] Li Y. Wang Y. Wan D. Li B. Zhang P. Wang H. (2020). Pilot-scale application of sulfur-limestone autotrophic denitrification biofilter for municipal tailwater treatment: performance and microbial community structure. Bioresour. Technol. 300:122682. 10.1016/j.biortech.2019.122682 31901555

[B28] Liang B. Kang F. Yao S. Zhang K. Wang Y. Chang M.. (2022). Exploration and verification of the feasibility of the sulfur-based autotrophic denitrification integrated biomass-based heterotrophic denitrification systems for wastewater treatment: from feasibility to application. Chemosphere 287:131998. 10.1016/j.chemosphere.2021.131998 34450373

[B29] Lin Q. Luo A. Wang Y. Liang Z. Zhang Y. Yu C. (2022). Deciphering autotrophic and heterotrophic partial nitrification under salinity stress. Int. Biodeterior. Biodegrad. 174:105472. 10.1016/j.ibiod.2022.105472

[B30] Navada S. Vadstein O. Gaumet F. Tveten A.-K. Spanu C. Mikkelsen O.. (2020). Biofilms remember: osmotic stress priming as a microbial management strategy for improving salinity acclimation in nitrifying biofilms. Water Res. 176:115732. 10.1016/j.watres.2020.115732 32278921

[B31] Nuhogl A. Pekdemir T. Yildiz E. Keskinler B. (2002). Drinking water denitrification by a membrane bio-reactor. Water Res. 36, 1155–1166. 10.1016/S0043-1354(01)00344-X 11902772

[B32] Ohore O. E. Wei Y. Wang J. Wang Y. Ifon B. E. Liu W.. (2022). Vertical characterisation of phylogenetic divergence of microbial community structures, interaction, and sustainability in estuary and marine ecosystems. Sci. Total Environ. 851(Pt 2):158369. 10.1016/j.scitotenv.2022.158369 36049676

[B33] Pang Y. Wang J. (2021). Various electron donors for biological nitrate removal: a review. Sci. Total Environ. 794:148699. 10.1016/j.scitotenv.2021.148699 34214813

[B34] Petcharat T. Benjakul S. (2018). Effect of gellan incorporation on gel properties of bigeye snapper surimi. Food Hydrocoll. 77, 746–753. 10.1016/j.foodhyd.2017.11.016

[B35] Polizzi C. Gabriel D. Munz G. (2022). Successful sulphide-driven partial denitrification: Efficiency, stability and resilience in SRT-controlled conditions. Chemosphere 295:133936. 10.1016/j.chemosphere.2022.133936 35149015

[B36] Ren J. Bai X. Liu Y. Huang X. (2021). Simultaneous nitrification and aerobic denitrification by a novel isolated Ochrobactrum anthropi HND19. Bioresour. Technol. 340:125582. 10.1016/j.biortech.2021.125582 34332445

[B37] Robertson E. K. Roberts K. L. Burdorf L. D. W. Cook P. Thamdrup B. (2016). Dissimilatory nitrate reduction to ammonium coupled to Fe(II) oxidation in sediments of a periodically hypoxic estuary. Limnol. Oceanogr. 61, 365–381. 10.1002/lno.10220

[B38] Shen Z. Xie L. Lyu C. Xu P. Yuan Y. Li X.. (2023). Effects of salinity on nitrite and elemental sulfur accumulation in a double short-cut sulfur autotrophic denitrification process. Bioresour. Technol. 369:128432. 10.1016/j.biortech.2022.128432 36473582

[B39] Shi J. Xu C. Han Y. Han H. (2020). Enhanced anaerobic degradation of nitrogen heterocyclic compounds with methanol, sodium citrate, chlorella, spirulina, and carboxymethylcellulose as co-metabolic substances. J. Hazard. Mater. 384:121496. 10.1016/j.jhazmat.2019.121496 31679892

[B40] Si Z. Song X. Wang Y. Cao X. Wang Y. Zhao Y.. (2021). Natural pyrite improves nitrate removal in constructed wetlands and makes wetland a sink for phosphorus in cold climates. J. Clean. Prod. 280:124304. 10.1016/j.jclepro.2020.124304

[B41] Song W. Ji J. IOP Publishing (2020). “Research on the effects of salinity on the microbial community of novel constructed wetland,” in 2020 6th International Conference on Energy Materials and Environment Engineering, Vol, 508 (TIanjin).

[B42] Song X. McDonald J. Price W. E. Khan S. J. Hai F. I. Ngo H. H.. (2016). Effects of salinity build-up on the performance of an anaerobic membrane bioreactor regarding basic water quality parameters and removal of trace organic contaminants. Bioresour. Technol. 216, 399–405. 10.1016/j.biortech.2016.05.075 27262094

[B43] Sun Z. Li Y. Li M. Wang N. Liu J. Guo H.. (2022). Steel pickling rinse wastewater treatment by two-stage MABR system: reactor performance, extracellular polymeric substances (EPS) and microbial community. Chemosphere 299:134402. 10.1016/j.chemosphere.2022.134402 35337819

[B44] Tan X. Acquah I. Liu H. Li W. Tan S. (2019). A critical review on saline wastewater treatment by membrane bioreactor (MBR) from a microbial perspective. Chemosphere 220, 1150–1162. 10.1016/j.chemosphere.2019.01.027 33395802

[B45] Tian T. Yu H. Q. (2020). Denitrification with non-organic electron donor for treating low C/N ratio wastewaters. Bioresour. Technol. 299:122686. 10.1016/j.biortech.2019.122686 31902635

[B46] Wang J. Zhou J. Wang Y. Wen Y. He L. He Q. (2020). Efficient nitrogen removal in a modified sequencing batch biofilm reactor treating hypersaline mustard tuber wastewater: the potential multiple pathways and key microorganisms. Water Res. 177:115734. 10.1016/j.watres.2020.115734 32278165

[B47] Wang S. Cheng H. Zhang H. Su S. Sun Y. (2021). Sulfur autotrophic denitrification filter and heterotrophic denitrification filter: comparison on denitrification performance, hydrodynamic characteristics and operating cost. Environ. Res. 197:111029. 10.1016/j.envres.2021.111029 33744267

[B48] Wang S. S. Jiang L. J. Hu Q. T. Cui L. Zhu B. T. Fu X. T.. (2021). Characterization of Sulfurimonas hydrogeniphila sp. nov., a novel bacterium predominant in deep-sea hydrothermal vents and comparative genomic analyses of the genus *Sulfurimonas*. Front. Microbiol. 12:626705. 10.3389/fmicb.2021.626705 33717015 PMC7952632

[B49] Wang Z. Gao M. Wang Z. She Z. Chang Q. Sun C.. (2013). Effect of salinity on extracellular polymeric substances of activated sludge from an anoxic-aerobic sequencing batch reactor. Chemosphere 93, 2789–2795. 10.1016/j.chemosphere.2013.09.038 24134890

[B50] Xu N. Tan G. Wang H. Gai X. (2016). Effect of biochar additions to soil on nitrogen leaching, microbial biomass and bacterial community structure. Eur. J. Soil Biol. 74, 1–8. 10.1016/j.ejsobi.2016.02.004

[B51] Xu S. Zhang F. Jiang Y. Zhang K. (2022). Characterization of a new heterotrophic nitrification bacterium *Pseudomonas* sp. strain JQ170 and functional identification of nap gene in nitrite production. Sci. Total Environ. 806:150556. 10.1016/j.scitotenv.2021.150556 34582850

[B52] Yan L. Zheng Y. Chen W. Liu S. Yin M. Jiang J.. (2022). Step feed mode synergistic mixed carbon source to improve sequencing batch reactor simultaneous nitrification and denitrification efficiency of domestic wastewater treatment. Bioresour. Technol. 358:127440. 10.1016/j.biortech.2022.127440 35680088

[B53] Yuan Z. Chen Y. Zhang M. Qin Y. Zhang M. Mao P.. (2022). Efficient nitrite accumulation and elemental sulfur recovery in partial sulfide autotrophic denitrification system: insights of seeding sludge, S/N ratio and flocculation strategy. Chemosphere 288 (Pt 2):132388. 10.1016/j.chemosphere.2021.132388 34695485

[B54] Zhang C. Gao F. Wu Y. Xu G. Liu H. Zhang H.. (2022). Small-sized salt-tolerant denitrifying and phosphorus removal aerobic granular sludge cultivated with mariculture waste solids to treat synthetic mariculture wastewater. Biochem. Eng. J. 181:108396. 10.1016/j.bej.2022.108396

[B55] Zhang M. Wang Z. J. Huang J. C. Sun S. Cui X. Zhou W.. (2021). Salinity-driven nitrogen removal and its quantitative molecular mechanisms in artificial tidal wetlands. Water Res. 202:117446. 10.1016/j.watres.2021.117446 34314924

[B56] Zhang Y. Hou K. Qian H. Gao Y. Fang Y. Tang S.. (2023). Natural-human driving factors of groundwater salinization in a long-term irrigation area. Environ. Res. 220:115178. 10.1016/j.envres.2022.115178 36584846

[B57] Zhou Y. Chen F. Chen N. Peng T. Dong S. Feng C. (2021). Denitrification performance and mechanism of biofilter constructed with sulfur autotrophic denitrification composite filler in engineering application. Bioresour. Technol. 340:125699. 10.1016/j.biortech.2021.125699 34391190

[B58] Zhu J. You H. Li Z. Xie B. Chen H. Ding Y.. (2022). Comparison on the photogranules formation and microbial community shift between the batch and continuous-flow mode for the high saline wastewater treatment. Chem. Eng. J. 446:137284. 10.1016/j.cej.2022.137284

[B59] Zhu Z. Zhou H. Zou J. Wang J. (2023). Effect of salinity on the denitrification of the sulfur-based autotrophic denitrification system. Water Cycle 4, 95–103. 10.1016/j.watcyc.2023.05.001

